# Supplementary material for: Rare Pediatric Cerebellar High-Grade Gliomas Mimic Medulloblastomas Histologically and Transcriptomically and Show p53 Mutations
Source: Cancers (Basel). 2024 Jan 4;16(1):232. doi: 10.3390/cancers16010232 (PMC10778382; doi:10.3390/cancers16010232)
Supplement: Supplementary file 1 [file cancers-16-00232-s001.zip › Supplementary Tables.pdf]

Table S1. Clinical features of the five cases.

Case	Sex	Age	Symptoms	MRI	Treatment	Outcome
1	M	13	Headache, vomiting	4th ventricular tumor	GTR → CSI → Chemo	NED (6m)
2	F	13	Headache, diplopia	L CB/CPA lesion	STR → CSI + carbo/VCR → Chemo x 4 cycles → PD	DOD (12m)
3	M	9	ALL survivor, history of cranial RT, headache, weakness, imbalance	R CB/CPA lesion	GTR → CSI → Chemo → PD	DOD (9m)
4	F	9	LLL weakness, imbalance	L CB/CPA lesion	GTR → Chemo x 1 cycle → PD 4 months from 1st surgery → second GTR	DOD (12m)
5	M	10	Headache, dizziness, vomiting	CB lesion	GTR	DOD (5m)

LLL, left lower limb weakness; ALL, acute lymphoblastic leukaemia; CB, cerebellum; CPA, cerebellopontine angle; STR, subtotal resection; GTR, gross total resection; CSI : craniospinal irradiation; PD, progressive disease; DOD, died of disease; NED, no evidence of disease.

Table S2. List of genes used in target sequencing panel.

ABCB1	ABCC4	ABCG2	ABL1	ABL2	ACVR1B	AKT1	AKT2	AKT3	ALK	ALOX12B	AMER1	APC	APCDD1	AR	ARAF
ARFRP1	ARID1A	ARID1B	ARID2	ASXL1	ATM	ATR	ATRX	AURKA	AURKB	AXIN1	AXL	BACH1	BAP1	BARD1	BCL2
BCL2A1	BCL2L1	BCL2L2	BCL6	BCOR	BCORL1	BLM	BMPR1A	BRAF	BRCA1	BRCA2	BRD4	BRIP1	BTG1	C11orf30	CARD11
CASP8	CBFB	CBL	CCND1	CCND2	CCND3	CCNE1	CD274	CD79A	CD79B	CDC73	CDH1	CDH2	CDH20	CDH5	CDK12
CDK4	CDK6	CDK8	CDKN1A	CDKN1B	CDKN2A	CDKN2B	CDKN2C	CEBPA	CHD2	CHD4	CHEK1	CHEK2	CHUK	CIC	CRBN
CREBBP	CRKL	CSF1R	CTCF	CTNNA1	CTNNB1	CUL3	CUL4A	CYLD	CYP17A1	CYP2C8	CYP3A4	CYP3A5	DAXX	DDR1	DDR2
DICER1	DIS3	DNMT3A	DOT1L	DPYD	EGFR	EP300	EPCAM	EPHA3	EPHA5	EPHA6	EPHA7	EPHB1	EPHB4	EPHB6	ERBB2
ERBB3	ERBB4	ERCC2	ERCC4	ERG	ERRFI1	ESR1	EZH2	FAM175A	FAM46C	FANCA	FANCC	FANCD2	FANCE	FANCF	FANCG
FANCI	FANCL	FANCM	FAS	FAT1	FAT3	FBXW7	FCGR3A	FGF10	FGF12	FGF14	FGF19	FGF23	FGF3	FGF4	FGF6
FGF7	FGFR1	FGFR2	FGFR3	FGFR4	FH	FLCN	FLT1	FLT3	FLT4	FOXL2	FOXP1	FRS2	FUBP1	GABRA6	GALNT12
GATA1	GATA2	GATA3	GATA4	GATA6	GEN1	GID4	GLI1	GNA11	GNA13	GNAQ	GNAS	GPR124	GREM1	GRIN2A	GRM3
GSK3B	H3F3A	HGF	HIF1A	HLA-A	HOXB13	HRAS	HSD3B1	HSP90AA1	IDH1	IDH2	IGF1	IGF1R	IGF2	IGF2R	IKBKE
IKZF1	IL7R	INHBA	INPP4B	INSR	IKBKE	IKZF1	IL7R	INHBA	INPP4B	INSR	INSRR	IRF2	IRF4	IRS2	JAK1
JAK2	JAK3	JUN	KAT6A	KDM5A	KDM5C	KDM6A	KDR	KEAP1	KEL	KIT	KLHL6	KMT2A	KMT2C	KMT2D	KRAS
LMO1	LRP1B	LRP2	LRP6	LRRK2	LTK	LYN	LZTR1	MAGI2	MAP2K1	MAP2K2	MAP2K4	MAP3K1	MAP3K13	MAP3K9	MAPK1
MAPK3	MCL1	MDM2	MDM4	MED12	MEF2B	MEN1	MERTK	MET	MITF	MKNK1	MKNK2	MLH1	MPL	MRE11A	MSH2
MSH6	MST1R	MTHFR	MTOR	MUTYH	MYC	MYCL	MYCN	MYD88	NBN	NCOR1	NF1	NF2	NFE2L2	NFKBIA	NKX2-1
NOTCH1	NOTCH2	NOTCH3	NOTCH4	NPM1	NRAS	NSD1	NTRK1	NTRK2	NTRK3	NUDT1	NUP93	PAK6	PAK7	PALB2	PARK2
PARP1	PARP2	PARP3	PARP4	PAX5	PBRM1	PDCD1LG2	PDGFRA	PDGFRB	PDK1	PHLPP2	PIK3C2B	PIK3C2G	PIK3C3	PIK3CA	PIK3CB
PIK3CG	PIK3R1	PIK3R2	PLCG2	PMS2	PNRC1	POLD1	POLE	PPARG	PPP2R1A	PRDM1	PREX2	PRKAR1A	PRKCI	PRKDC	PRSS1
PRSS8	PTCH1	PTCH2	PTEN	PTK2	PTK2B	PTPN11	PTPRD	QKI	RAC1	RAD50	RAD51	RAD51B	RAD51C	RAD51D	RAD52
RAD54L	RAF1	RANBP2	RARA	RB1	RBM10	REL	RET	RhoA	RICTOR	RNF43	ROS1	RPA1	RPTOR	RUNX1	RUNX1T1
SDHA	SDHB	SDHC	SDHD	SETD2	SF3B1	SH2B3	SLC22A2	SLIT2	SMAD2	SMAD3	SMAD4	SMARCA4	SMARCB1	SMARCD1	SMO
SNCAIP	SOCS1	SOX10	SOX2	SOX9	SPEN	SPOP	SPTA1	SRC	STAG2	STAT3	STAT4	STK11	SUFU	SULT1A1	SYK
TAF1	TBX3	TEK	TERT ^	TET2	TGFBR2	TIPARP	TNF	TNFAIP3	TNFRSF14	TNKS	TNKS2	TOE1	TOP1	TOP2A	TP53
TP53BP1	TPMT	TRRAP	TSC1	TSC2	TSHR	TYRO3	U2AF1	UGT1A1	UGT1A7	UMPS	VEGFA	VHL	WISP3	WT1	XPO1
XRCC2	XRCC3	ZBTB2	ZNF217	ZNF703	ZNRF3										

^ promoter region
