# Supplementary material for: Rare Pediatric Cerebellar High-Grade Gliomas Mimic Medulloblastomas Histologically and Transcriptomically and Show p53 Mutations
Source: Cancers (Basel). 2024 Jan 4;16(1):232. doi: 10.3390/cancers16010232 (PMC10778382; doi:10.3390/cancers16010232)
Supplement: Supplementary file 1 [file cancers-16-00232-s001.zip › Supplementary Figure.pdf]

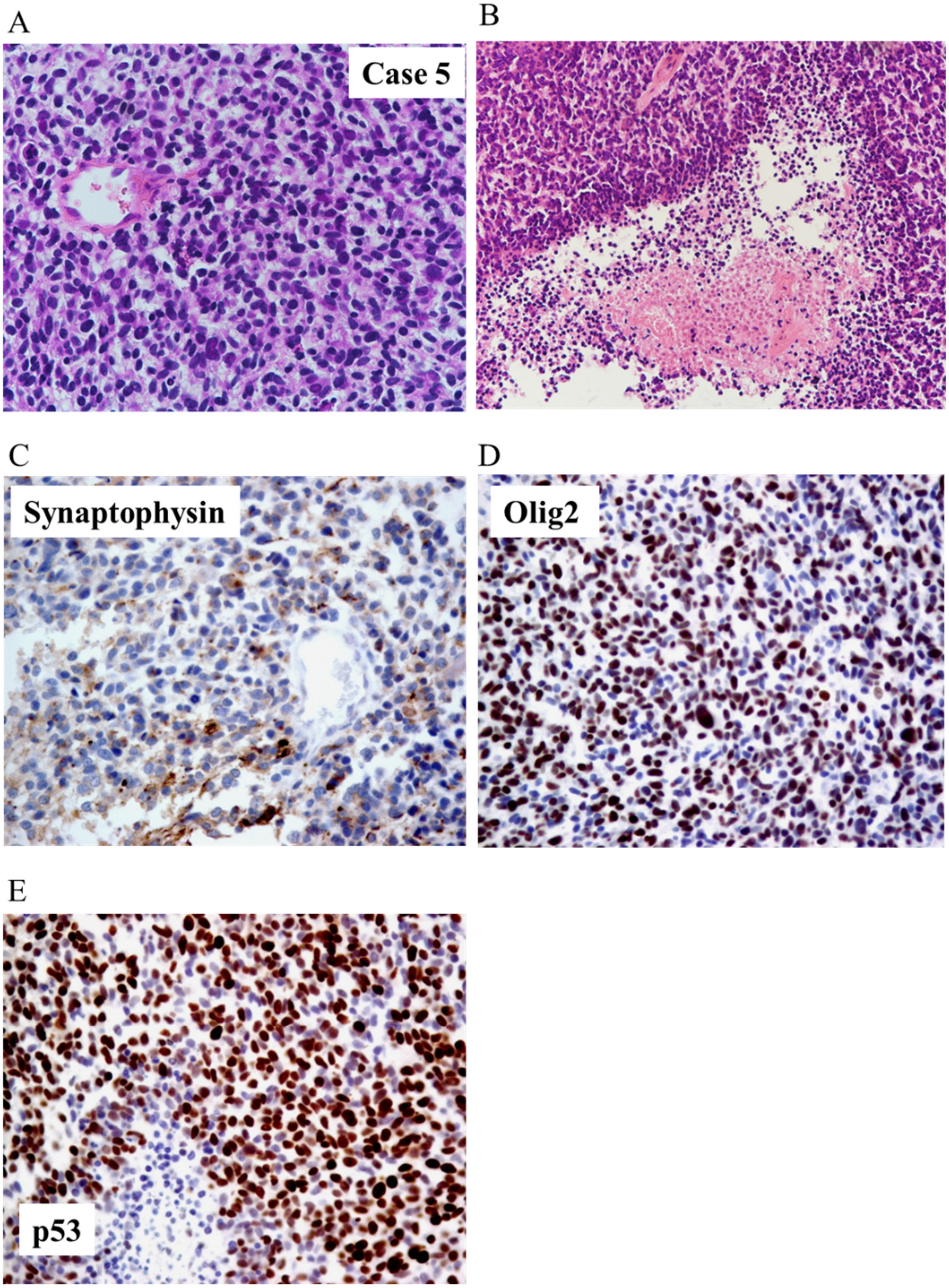

Figure S1. (A–E) Case 5. MR images are not available. (A) H&E shows sheets of embryonal cells with scanty cytoplasm, similar to the other cases. (B) necrotic foci are seen (×200). Immunostaining for (C) synaptophysin (×400), (D) Olig2 (×200), and (E) p53 (×200). Tumor cells were also focally positive for GFAP and negative for NeuN, positive for INI-1, and showed high labeling for Ki67.
